# Intramedullary nailing at different distal tibial fracture levels: A biomechanical study

**DOI:** 10.1097/MD.0000000000038353

**Published:** 2024-05-31

**Authors:** Ortac Guran, Ramadan Ozmanevra, Resit Bugra Husemoglu, Hasan Havitcioglu, Ozenc Altinoz

**Affiliations:** a Sancaktepe Şehit Prof. Dr. Ilhan Varank Training and Research Hospital, Istanbul, Turkey; b Cyprus International University, Nicosia, Cyprus; c Department of Biomechanics, Institute of Health Sciences, Dokuz Eylul University, Izmir, Turkey; d Department of Orthopedics and Traumatology, Dokuz Eylul University, Izmir, Turkey; e Primemed Clinic, Kyrenia, Cyprus.

**Keywords:** biomechanics, distal tibia, fracture, intramedullary nailing

## Abstract

**Background:**

Distal tibial fractures remains a significant challenge in orthopedic trauma surgery. As the fracture level approaches the joint, alternative fixation options instead of intramedullary nailing (IMN) come to the fore. The present study aimed to assess the biomechanical stability of IMN at different distal tibial fracture levels and the number of locking screws required.

**Methods:**

Using a total of 21 sawbone models, 3 different tibial fracture levels (3, 4.5, and 6 cm proximally to the talocrural joint) were created and the fractures were fixed using 2, 3, or 4 distal locking screws. A single compression force at a speed of 30 mm/min with a maximum force of 800 Newton and a cyclic compression force of 60 cycles at a speed of 60 mm/min was applied to all tibia models. The applied weight and displacements from the fracture lines were recorded and evaluated.

**Results:**

There was no statistically significant difference in fixation with 2 distal locking screws in groups 1, 2, and 3 (single test *P* =.9689) (cyclic test *P* =.8050). Therefore, if 2 distal screws are used, the fracture level does not affect the strength of fixation. In fractures located 6 cm proximal to the talocrural joint, all 4 holes of the nail can be used to insert screws, which provides a stronger fixation. When 2 screws are used, a statistically weaker fixation is obtained than with 3 or 4 screws. However, there is no significant difference between using 3 or 4 screws.

**Conclusion:**

Our findings support the use of IMN with 2 distal locking screws as a viable option for the management of distal tibial fractures. We found that it provides sufficient fixation regardless of the fracture level, suggesting that there is no need to choose an alternative fixation technique due to concerns of inadequate fixation as the fracture line moves distally. In cases where more stable fixation is desired, an additional locking screw can be used, but the potential increase in procedure and fluoroscopy time should be considered.

## 1. Introduction

The treatment of tibia fractures involves various methods such as casting, anatomical plates, nails, or external fixators. Factors such as the general condition of the patient, the type and location of the fracture, the soft tissue coverage, and the experience of the surgeon are all taken into consideration in determining the appropriate treatment strategy. The location of the fracture is one of the most important variables among these factors. While intramedullary nailing (IMN) is considered the gold standard in midshaft fractures, there are concerns about its use in proximal or distal tibial fractures.^[1]^

Distal metaphyseal fractures account for almost 18% of all tibia fractures, and surgical treatment, such as plate fixation or IMN, is the preferred approach as it enables early mobilization and results in better bone structure alignment.^[2–4]^ Plate fixation may be preferred when the fracture line approaches distally, as it restores tibia alignment and enables early rehabilitation for patients. However, the anatomical conditions of this area, including poor blood supply and thin soft tissue coverage, can lead to wound problems and higher reoperation rates.^[5]^ IMN causes less soft tissue damage and preserves the blood supply of the joint in distal tibia fractures compared to plate fixation.^[6]^ IMN may be a better option if there is sufficient bone stock for distal locking screws. With the evolution of nail designs, the distal screw holes are now positioned at the most distal points on the nail, and oblique locking is also possible in addition to anteroposterior and lateral locking screws. Secure fixation is aimed with locking screws in different planes, as the nail-bone contact decreases as it moves distally on the tibia surface. However, the safe application of IMN in distal tibial fractures is still uncertain. A recent computer-assisted search of the scientific literature revealed no biomechanical studies comparing different distal tibia fracture levels and instabilities in sawbones stabilized with intramedullary nails. Therefore, the purpose of this study is to determine the most distal point where IMN is a safe alternative for fixation.

## 2. Material and methods

In this study, 21 sawbone models were used. The Poisson ratio for materials used in sawbones can vary depending on the specific composition, but it typically ranges from 0.25 to 0.35. Sawbones materials often have an elastic limit or yield strength in the range of 50 to 100 MPa (Megapascals). The breaking stress or ultimate tensile strength (UTS) for sawbones materials is typically around 100 to 150 MPa. The tensile stress at yield (yield strength) for sawbones materials ranges from 30 to 70 MPa. The compressive strength of sawbone materials is usually in the range of 100 to 200 MPa.

**Figure 1.**
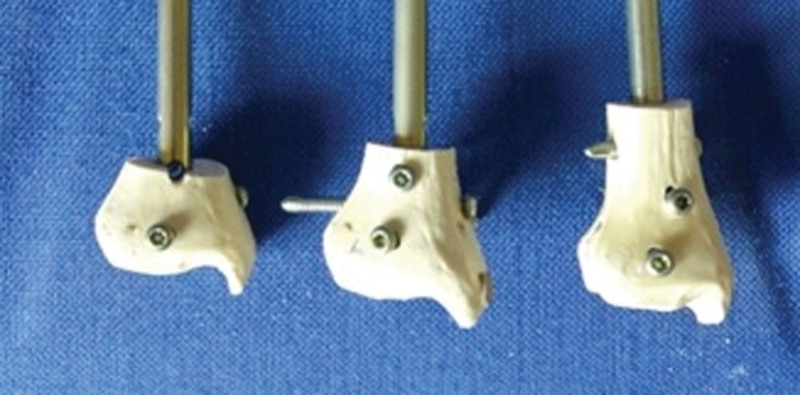
Three different fracture models 3 cm, 4.5 cm, and 6 cm to tibial plafond.

Three different fracture levels were created in 21 tibia sawbone models: Group 1 (3 cm), Group 2 (4.5 cm), and Group 3 (6 cm proximally to the talocrural joint) (Fig. 1). Depending on the fracture levels, different numbers of distal locking screws were used: 2 screws in Group 1 (Group 1-2S), 3 screws in Group 2 (Group 2-3S), and 2, 3, or 4 screws in Group 3 (Group 3-2S, Group 3-3S, Group 3-4S).

**Figure 2.**
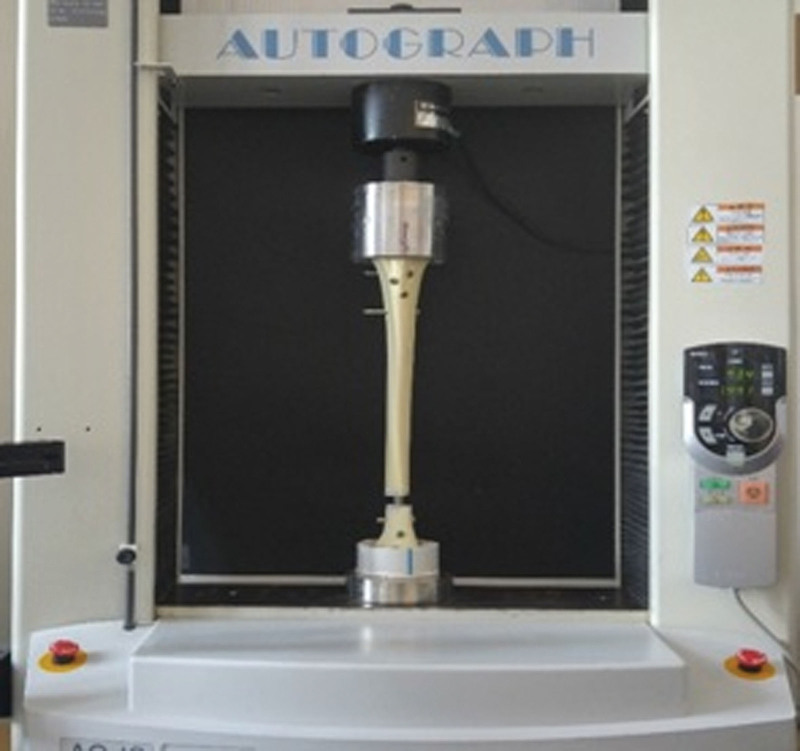
5Hz/ 5kN electro-mechanical actuator for biomechanical testing.

A 10mm wide transverse osteotomy at the transition of segment 43 was chosen as the fracture model according to the Muller AO classification.^[7,8]^ The sawbones distal tibia fracture models were fixed with a 9mm Expert Tibial Nail (Synthes®, Switzerland) (Fig. 2) using 3 proximal locking screws and 2, 3, or 4 distal locking screws. The nailing system is manufactured from Ti-6Al-7Nb. The Young modulus of this system (Titanium-6% Aluminum-7% Niobium alloy (Ti-6Al-7Nb)) typically ranges from approximately 95 to 110 Gigapascals. The Poisson ratio for Ti-6Al-7Nb alloy is typically around 0.32 to 0.34 and the elastic limit of Ti-6Al-7Nb alloy is approximately 500 MPa (Megapascals). The breaking stress or UTS of Ti-6Al-7Nb alloy ranges from 800 to 1000 MPa while the tensile stress at yield (yield strength) for Ti-6Al-7Nb alloy is typically around 550 to 650 MPa. The compressive strength of Ti-6Al-7Nb alloy is approximately 1100 to 1300 MPa.

**Figure 3.**
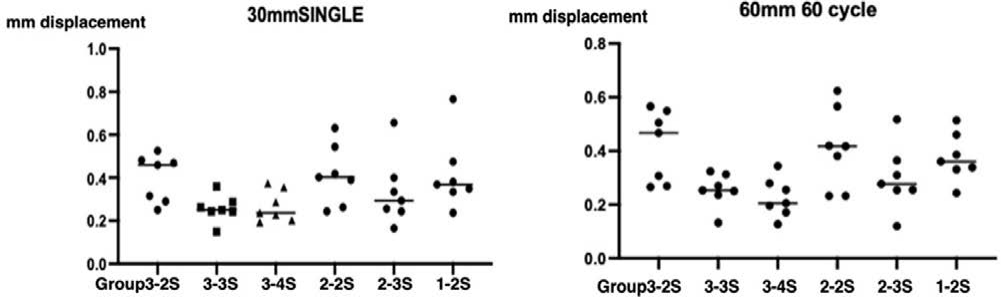
Individual values of 30 mm single (A) and 60 mm 60 cycles (B) groups. Horizontal bars indicate the mean values.

The nail was placed on the tibial plateau in the anterior-posterior projection towards the lateral intercondylar tubercle and in line with the axis of the medullary canal. The nail was removed for the osteotomy procedure and reinserted after bone resection. All surgeries were performed by a single experienced trauma surgeon. Both ends of the tibia were placed in polymethyl methacrylate for insertion into the testing machine for biomechanical loading. All tibia models with IMN were transferred to Dokuz Eylul University, Department of Biomechanics for biomechanical testing. A single compression force at a speed of 30mm/min with a maximum force of 800 Newton and a cyclic compression force of 60 cycles at a speed of 60mm/min was applied to all tibia models. Video extensometer markers were placed 5mm distally and proximally to the fracture line to assess the regional displacement of the fracture line. The applied weight and displacements were recorded in real time with the device software. All tests were carried out with an electromechanical actuator under axial load (Fig. 3).

Statistical analysis was conducted using GraphPad Prism 9.3.1. Both the Single Test (30mm single) and Cyclic Test (60mm 60 cycles) were carried out on study groups. The Cyclic Test involved averaging 3 replicates (cycles 58–60). Data were checked for normal distribution using the Shapiro–Wilk test. Mean and standard deviation were used for data with normal distribution, while frequency and percentage values were used for categorical variables. In addition, the mean ± standard deviation (SD) was used for continuous variables. Student *t* test was used to compare 2 independent groups, and the One-Way ANOVA test was used to compare 3 independent groups. Post hoc analysis was performed using Tukey test. A *P* value of <.05 was considered statistically significant in all tests.

## 3. Results

Regional displacement of the fracture line of study groups in the single test and cyclic test are documented (Table 1).

**Table 1:** The displacement values of the fracture line in single and cyclic tests.

Single test (30 mm)	Displacement (mm)
Group3-4S	0.2688 ± 0.07287
Group3-3S	0.2565 ± 0.06235
Group3-2S	0.3987 ± 0.1096
Group2-3S	0.3357 ± 0.1594
Group2-2S	0.4134 ± 0.1373
Group1-2S	0.4161 ± 0.1667

Cyclic test (60 mm 60 cycles)	Displacement (mm)
Group3-4S	0.2253 ± 0.07269
Group3-3S	0.2539 ± 0.06290
Group3-2S	0.4183 ± 0.1333
Group2-3S	0.2996 ± 0.1213
Group2-2S	0.4101 ± 0.1495
Group1-2S	0.3760 ± 0.08903

*There was no statistically significant difference in fixation with 2 distal locking screws in groups 1, 2, and 3* (single test *P* = .9689) (cyclic test *P* = .8050). Therefore, if 2 distal screws are used, the fracture level does not affect the strength of fixation.

In the Single Test (30 mm), *Group 3-2S had a higher displacement value than Group 3-3S and Group 3-4S (P* = .0093 by one-way ANOVA; adjusted *P* values = .0142 and .0251 for comparisons with Group3-3S and Group3-4S, respectively, by Tukey test; Fig. 3A). No other significant difference was observed in the single test.

In the Cyclic Test (60mm 60 cycles), *Group3-2S had a higher displacement value than Group3-3S and Group3-4S (P* = .0026 by one-way ANOVA; adjusted *P* values = .0120 and .0035 for comparisons with Group3-3S and Group3-4S, respectively, by Tukey test; Fig. 3B). No other significant difference was observed in the cyclic test.

In distal tibial fractures located 6 cm proximal to the talocrural joint, all 4 holes of the nail can be used to insert screws, which provides a stronger fixation. When 2 screws are used, a statistically weaker fixation is obtained than with 3 or 4 screws. However, there is no significant difference between using 3 or 4 screws.

## 4. Discussion

Biological fixation of fractures has 2 advantages over rigid fixation: firstly, by generating a larger amount of cartilage, callus volume increases, reducing the risk of implant hardware failure due to early weight bearing; secondly, biological healing can tolerate the negative effects of instability caused by screw loosening, which occurs because of stress shielding.^[9,10]^ Evidence suggests that the mechanical conditions of the fracture gap, determined by fixation stability, can influence healing outcomes.^[11–13]^

Approximately 18% of all tibia fractures are distal tibia fractures, and their management remains a challenge.^[4,14]^ Numerous studies compare minimally invasive plate osteosynthesis (MIPO) and IMN for distal metaphyseal tibial fractures.^[15–17]^ Clinically, IMN may preferable to MIPO because it has a smaller impact on the surrounding soft tissues and vascularization, reducing the risk of related complications such as infection.^[18,19]^ Koksal et al concluded that IMN is a cost-effective and superior option in terms of union time and return to the market compared to MIPO in the treatment of extra-articular distal tibia fractures.^[20]^ Bleeker et al revealed that IMN offers faster bone union, quicker weight-bearing, and reduced surgery time while MIPO results in a lower risk of mal-union.^[21]^ The need for 2 distal locking screws in some fractures has been debated.^[22,23]^ George et al examined the impact of altering the position of a single distal locking screw in fractures of the distal third of the femur and found that rotational stability increases as the locking screw gets closer to the fracture.^[24]^ Moreover, a biplanar distal interlocking procedure is suggested to enhance bone union time as it provides greater stability.^[25]^ This biomechanical study aimed to assess the primary stability of tibial IMN at different distal tibial fracture levels (3, 4.5, and 6 cm proximally to the talocrural joint) fixed with 2, 3, or 4 distal locking screws.

Greenfeld et al established that double locking preserved compressive stiffness by 60% to 70% and torsional stiffness by 90% compared to triple locking in retrograde tibia nailing.^[26]^ Thus, the IMN with a distal double locking can be considered for far distal tibia fractures where nailing is preferred over plating. Accordingly, we found similar displacement values in single and cyclic tests at all 3 fracture levels fixed with 2 distal locking screws. Therefore, *we think that sufficient fixation can be achieved with 2 distal locking screws in IMN, regardless of the fracture level,* suggesting that there is no need to choose an alternative fixation technique due to concerns of inadequate fixation as the fracture line moves distally.

Several studies have been conducted to improve the stability of nails used for treating distal tibia fractures. Augat et al compared 3 different fixation methods biomechanically: conventional locking with 8- and 10-mm-diameter nails and angular stable locking with 8-mm nails.^[6]^ However, their hypothesis that angular stable interlocking would improve the mechanical performance of distal tibia fracture fixation was not confirmed. We used conventional locking and found that 2 distal locking screws provide adequate stability in all 3 distal tibial fracture levels.

In distal tibial fractures located 6 cm proximal to the talocrural joint, all 4 holes of the nail can be used to insert screws. A statistically stronger fixation is obtained when using 3 or 4 distal locking screws compared to 2 screws. However, the fixation strength of using 3 or 4 screws is similar. Therefore, more than 2 distal locking screws can be used if stronger fixation is desired, but surgeons should consider the potential extension of surgery and fluoroscopy duration due to the insertion of extra screws. Additionally, since anteroposterior distal tibial locking bolts lie close to the neurovascular bundle,^[27]^ using extra screws may increase the risk of damaging the neurovascular bundle.

Apart from axial loading, torsional forces could have also been investigated. This can be seen as one of the limitations of this study. Another limitation is that the study was conducted using sawbones, which may suggest that these results could be more valuable in clinical practice.

In recent years, studies related to biomaterials, biomedical engineering, and innovative technologies including finite element analysis and biomechanics have been increasing in orthopedics^[28,29]^ and also comparative studies are being conducted, especially in orthopedics, regarding fixation options.^[15,16,20,30]^ We believe that this biomechanical study we conducted will guide surgeons in clinical practice and shed light on future studies related to this topic.

## 5. Conclusion

We found that 2 distal locking screws provided adequate fixation at all 3 distal tibial fracture levels. Our findings support the use of IMN with 2 distal locking screws as a viable option for treating distal tibial fractures, offering advantages such as shorter union time, rapid weight bearing, and reduced risk of infection. In cases where more stable fixation is desired, an additional locking screw may be used, but the potential increase in procedure and fluoroscopy time, and the risk of damaging the neurovascular bundle should be considered.

## Author contributions

**Conceptualization:** Ortac Guran, Ramadan Ozmanevra, Resit Bugra Husemoglu, Hasan Havitcioglu.

**Data curation:** Ortac Guran, Resit Bugra Husemoglu, Hasan Havitcioglu.

**Formal analysis:** Ortac Guran, Resit Bugra Husemoglu, Hasan Havitcioglu.

**Funding acquisition:** Ortac Guran, Hasan Havitcioglu.

**Investigation:** Ortac Guran, Resit Bugra Husemoglu, Hasan Havitcioglu, Ozenc Altinoz.

**Methodology:** Ortac Guran, Ramadan Ozmanevra, Resit Bugra Husemoglu, Hasan Havitcioglu.

**Project administration:** Ortac Guran, Hasan Havitcioglu, Ozenc Altinoz.

**Resources:** Ortac Guran, Hasan Havitcioglu.

**Software:** Ortac Guran, Hasan Havitcioglu.

**Supervision:** Ortac Guran, Hasan Havitcioglu.

**Validation:** Ortac Guran, Hasan Havitcioglu.

**Visualization:** Ortac Guran, Hasan Havitcioglu, Ozenc Altinoz.

**Writing – original draft:** Ortac Guran, Ramadan Ozmanevra, Resit Bugra Husemoglu, Hasan Havitcioglu, Ozenc Altinoz.

**Writing – review & editing:** Ortac Guran, Ramadan Ozmanevra, Resit Bugra Husemoglu, Hasan Havitcioglu, Ozenc Altinoz.
